# Malnutrition in Amyotrophic Lateral Sclerosis: Insights from Morphofunctional Assessment and Global Leadership Initiative on Malnutrition Criteria

**DOI:** 10.3390/nu16162625

**Published:** 2024-08-09

**Authors:** María Teresa Zarco-Martín, Carmen Freire, María Carmen Andreo-López, Socorro Leyva-Martínez, María Luisa Fernández-Soto

**Affiliations:** 1 Endocrinology and Nutrition Unit, San Cecilio University Hospital, 18016 Granada, Spain; 2 Fundación para la Investigación Biosanitaria en Andalucía Oriental-Alejandro Otero (FIBAO), 18012 Granada, Spain; 3 Department of Legal Medicine, Toxicology and Physical Anthropology, University of Granada, 18006 Granada, Spain; 4 Instituto de Investigación Biosanitaria de Granada (Ibs. Granada), 18012 Granda, Spain; 5 CIBER de Epidemiología y Salud Pública, 28029 Madrid, Spain; 6 Department of Medicine, University of Granada, 18016 Granada, Spain

**Keywords:** ALS, malnutrition, phase angle, body composition

## Abstract

Amyotrophic Lateral Sclerosis (ALS) is a progressive neurodegenerative disease frequently accompanied by malnutrition due to weight loss, increased energy expenditure, and muscle mass loss. This study aimed to evaluate morphofunctional assessment tools as predictors of malnutrition and to investigate their relationship with muscle status and disease severity in ALS patients. A cross-sectional study was conducted with 45 ALS patients at the San Cecilio University Hospital in Granada. Malnutrition was assessed using the Global Leadership Initiative on Malnutrition (GLIM) criteria. Morphofunctional assessment was performed using Bioimpedance Vectorial Analysis (BIVA), handgrip strength (HGS), and Short Physical Performance Battery (SPPB). Malnutrition prevalence was 38% according to GLIM criteria. Significant differences were observed between malnourished and non-malnourished groups in age (70 ± 9 vs. 62 ± 10 years, *p* = 0.01), sex (female prevalence: 58.8% vs. 25.0%, *p* = 0.02), dysphagia prevalence (83% vs. 29%, *p* < 0.001), PEG/PRG use (35.3% vs. 3.6%, *p* = 0.01), and ALSFRS-R scores (30 ± 12 vs. 34 ± 12, *p* = 0.02). Malnourished patients had lower values in anthropometric measurements, muscle mass obtained by BIVA, and phase angle (PA) (4.05 ± 0.8° vs. 5.09 ± 0.8°, *p* < 0.001). No significant differences were found in muscle strength or functional status. PA showed significant correlations with muscle strength (r = 0.52, *p* < 0.001) and muscle mass measures (r = 0.48, *p* < 0.001). Moreover, PA was associated with poorer disease progression and physical performance. In our sample, BIVA metrics such as PA (<4.3°), SPA (<−0.8), body cell mass (<9.2 kg/m), and extracellular water (>49.75%) were identified as malnutrition risk factors. The study underscores the critical importance of comprehensive morphofunctional assessment and the use of advanced diagnostic criteria, for early identification and intervention in malnutrition among people with ALS. Further research is warranted to validate these findings and develop targeted nutritional strategies into routine clinical practice.

## 1. Introduction

Amyotrophic Lateral Sclerosis (ALS) is a progressive neurodegenerative disease characterized by the selective loss of motor neurons in the brain and spinal cord, leading to muscle weakness. The incidence of ALS is estimated to be about 2–3 cases per 100,000 people, with some variation across different regions [1]. The most common symptoms are muscle weakness, fatigue, atrophy, fasciculation, dysarthria, dysphagia, sialorrhea, and emotional instability [2].

In addition, ALS patients are at high risk of malnutrition due to weight loss, eating difficulties, increased energy needs, and muscle mass loss, affecting their quality of life and survival [3,4]. In fact, nutritional status is considered a significant prognostic factor and, specifically, muscle mass loss also aggravates the loss of spinal motor neurons [5].

Malnutrition prevalence in ALS patients ranges from 15 to 55% [6]. Its diagnosis is particularly challenging due to the heterogeneity in criteria and tools, leading to ambiguous assessment of nutritional state and the implementation of effective nutritional therapy [7].

Traditionally, BMI has been used for the diagnosis of malnutrition and has been considered as a clinical outcome predictor [8]; however, it does not allow us to detect changes in body composition or muscle mass loss. Some tools used in morphofunctional assessment, such as bioelectrical impedance analysis, have been described in ALS patients [9]. However, there is a lack of evidence on the use of functional and muscle assessment tools in this population. Therefore, there is a need of a holistic approach of these patients, including a morphofunctional assessment. This approach involves assessing body composition and muscle and functional state, focusing on both the quantity and quality of the body compartments for nutritional and physical intervention [10].

The hypothesis of this study is that morphofunctional assessment tools, including BIVA, HGS, and SPPB, can provide accurate and early identification of malnutrition in ALS patients when used alongside the GLIM criteria.

This study aims to evaluate morphofunctional assessment tools as predictors of malnutrition and their relationship with muscle status and disease progression in ALS patients.

## 2. Materials and Methods

### 2.1. Study Design and Population

A single-center cross-sectional observational study of people with ALS who attended the nutrition consultation in the multidisciplinary team of ALS (UMELA) at San Cecilio University Hospital in Granada was conducted between March 2022 and January 2023.

The sample size was determined using an a priori power analysis with G*Power. A power level of 0.80 (80%) and an alpha level of 0.05 were set as thresholds for detecting significant differences and associations. The analysis indicated that a minimum of 36 patients was required to achieve sufficient power.

A total of 45 patients were included in the study; 29 patients (64%) were attended to for the first time at the time of data collection during UMELA. The inclusion criteria were patients diagnosed with ALS according to El Escorial criteria, who were aged over 18 years old, and who agreed to participate in the study and signed the informed consent. The exclusion criteria were people with neurodegenerative diseases other than ALS. The study was approved by the Biomedical Research Ethics Committee of Granada (approval no. 1770-N-21), approval date: 25 February 2022.

### 2.2. Clinical Variables

Clinical variables included data on sex (male/female), age (years), evolution of the disease since ALS diagnosis (months), dysphagia diagnosed by the volume and viscosity test (MECV-V) [11], use of percutaneous endoscopic or radiologic gastrostomy (PEG or PRG), type of symptomatology onset (bulbar/spinal), and use of non-invasive mechanic ventilation (NIMV).

#### 2.2.1. Anthropometric Measurements

Habitual weight (kg) in the last 6–12 months was reported by the patients. Actual weight (kg) was assessed using a scale (SECA, Birmingham, UK). Height (m) was obtained using a stadiometer (SECA, Birmingham, UK). Weight loss (habitual weight-actual weight/habitual weight × 100) and body mass index (actual weight/height × height (kg/m^2^) were calculated.

Arm circumference (AC) was obtained at the midpoint between the acromion and olecranon with a measuring tape (SECA 201, Birmingham, UK), in cm. Triceps skinfold (TS) was obtained with a skinfold caliper (Holtain LTD, Crymych, UK), in mm. Arm muscle circumference (AMC) was calculated with the formula AC (cm) − (0.314 × TS (mm)).

Calf circumference (CC) was obtained at the widest section of the calf area with a measuring tap, in cm. We calculated the appendicular skeletal mass index (ASMI) with a predictive equation using calf circumference (in cm), age (in years), height (in m), and sex [12].

#### 2.2.2. Phase Angle and Body Composition Parameters

Phase angle and body composition analyses were obtained using Nutrilab^®^, a 50 kHz phase-sensitive impedance analyzer (Akern, Florence, Italy [13]). The PA was expressed in degrees as arctan (Xc/R) × (180°/p). An individual standardized PA value (SPA) was determined by adjusting it by sex and age. Data obtained using BIVA for body composition were categorized as fat-free mass (FFM/height, kg/m), fat mass (FM/height, kg/m), total body water (TBW/height, kg/m), extracellular water (% ECW of TBW), body cellular mass (BCM/height, kg/m), skeletal muscle mass index (SMI, kg/m^2^), and appendicular skeletal muscle mass (ASMM, kg), obtained from predictive equations [14,15]. Normality values from Nutrilab^®^ were used [16].

BIVA was conducted following established guidelines to ensure accuracy and reproducibility. Participants were positioned in a supine position with their limbs slightly apart to prevent skin contact, which could interfere with the electrical impedance measurement and to ensure stability. A five-minute rest period in the supine position was ensured prior to measurements to minimize the impact of fluid shifts caused by changes in posture. The BIVA device was calibrated daily according to the manufacturer’s instructions, and the accuracy of the BIVA measurements was verified using a precision circuit provided by the manufacturer.

#### 2.2.3. Muscle Strength

Muscle strength was assessed using an adult dynamometer (Jamar handgrip dynamometry, Asimow Engineering Co., Los Angeles, CA, USA) and measurements were performed in the dominant limb, repeated on three occasions, and the highest value was used to represent HGS.

To classify normality, we used the cut-off points proposed by the EWGSOP2 (males > 27 kg and females > 16 kg) [17].

#### 2.2.4. Biochemical Analysis

Serum albumin and high-sensitivity C-reactive protein (hs-CRP) levels were measured using standard laboratory techniques.

### 2.3. Physical Performance

Physical performance was analyzed using the SPPB [18], which includes 3 domains: balance, walking speed, and getting up from and sitting down on a chair 5 times.

In the balance test, the participant tries to hold 3 positions: feet together, semi-tandem, and tandem for 10 s each. These subtests follow a hierarchical sequence. In the walking speed test, the participant walks a distance of 4 m at his/her usual pace. This test is performed and the time is recorded in seconds. Finally, in the stand up and sit down on a chair 5 times test, the participant stands up and sits down on a chair 5 times, as fast as possible, and the total time taken is recorded.

Each test is scored from 0 (worst performance) to 4 (best performance). A total score is obtained which is the sum of the 3 tests and ranges from 0 to 12. The cut-off point to assess poor physical performance is ≤8 [17].

### 2.4. Malnutrition Disease Related Diagnosis

Malnutrition diagnosis was based on the GLIM criteria [19] whereby patients need to meet the sum of at least two criteria: a phenotypic criterion, i.e., weight loss, BMI, and the decrease in muscle mass; and an etiological criterion decrease in the intake of the requirements and the presence of inflammatory biomarkers. To assess muscle mass, we used ASMI calculated using a predictive equation. To classify normality, the cut-off points used were males > 7 kg/m^2^ and females > 6 kg/m^2^. The severity of malnutrition was defined: moderate cases included a BMI < 20 kg/m^2^ at age < 70 or BMI ≤ 22 kg/m^2^ at age ≥ 70, weight loss between 5 and 10% in the last 6 months. Severe cases included a BMI < 18.5 kg/m^2^ at age < 70 or BMI ≤ 20 kg/m^2^ at age ≥ 70, weight loss ≥ 10% in the last 6 months.

### 2.5. Disease Progression

The ALSFRS-R (Revised Amyotrophic Lateral Sclerosis Functional Rating Scale) score was used to assess ALS severity [20]. It is 12-item scale that assesses activities of daily living affected during the course of the disease. The total score ranges from 0 to 48, in which higher scores indicate better physical function [21].

### 2.6. Statistical Analysis

Data statistical analyses were carried out using IBM SPSS 25.0 (IBM, New York, NY, USA), and graphic representation was performed using R v.3.5.1 software (Integrated Development for R. RStudio, PBC, Boston, MA, USA).

Normality of the distribution of quantitative variables was verified using the Shapiro–Wilk test. Quantitative variables are presented as the mean and standard deviation, and differences between paired observations according to the nutritional status (malnutrition diagnosis or not) were determined using Student’s t-test (or the Wilcoxon test in the absence of normality). The qualitative variables are described as proportions, and the differences between groups were analyzed via the Chi-square test, using Fisher’s exact test when necessary. Pearson correlation coefficients between quantitative variables were obtained.

Logistic regression analysis was used to calculate the association between malnutrition and morphofunctional measurements, using as independent variables those with significant differences in Student’s *t*-test (*p* < 0.05) between malnourished and well-nourished groups, and clinical relevance. The model was adjusted for sex and age due to the possible interference of these parameters with the variables of interest owing to physiological factors.

The predictive capability of morphofunctional variables was assessed using the receiver operating characteristic (ROC) curves and the area under the curve (AUC). Statistical significance was set at *p* < 0.05.

## 3. Results

### 3.1. General Characterization of the Population Study

A total of 45 patients were included in the study, 28 (62.2%) males and 17 (37.8%) females. Mean age of the participants was 65 ± 10 years. Mean disease evolution time was 32 ± 27 months. Dysphagia prevalence was 49%. A total of 16% of the patients used PRG/PEG and 20% NIMV. A total of 44.4% had bulbar onset, 40% spinal onset, 11.1% had primary lateral sclerosis, and 4.4% had flail arm. The ALSFRS-R median was 32.

According to GLIM criteria, 17 patients (38%) were malnourished, of which 24.4% (11) were classified as moderate malnutrition and 6 (13.3%) as severe malnutrition.

Table 1 shows that malnourished ALS patients had significatively higher age, female prevalence, dysphagia diagnosis, and PEG/PRG use, and a lower ALSFRS-R.

No significant differences were found between non-malnourished and malnourished groups in serum albumin levels (4.2 ± 0.4 mg/dL vs. 4.0 ± 0.4 mg/dL, *p* = 0.2) and hs-CRP (4.5 ± 5.2 mg/L vs. 4.1 ± 3.9 mg/L, *p* = 0.8).

### 3.2. Morphofunctional Status

Morphofunctional assessment variables and differences between patients with and without a diagnosis of malnutrition are shown in Table 2. The mean percentage of weight loss (WL) was 9.5 ± 8.2%. There were significant differences between groups, with higher values of % WL in the malnourished group (11.8 ± 9.6% vs. 5.8 ± 2.5%, *p* = 0.03) over the last 6–12 months.

There was a statistically significant difference in anthropometric parameters, showing lower values in the malnourished group for all measurements except TS and CC. Significant differences were found in mean BIVA variables based on malnutrition diagnosis. Specifically, the malnutrition group showed notably lower values of PA, TBW, FFM, FM, BCM, ASMM, and SM, alongside higher values of Rz, ECW, and ECW/ICW.

Regarding HGS values, 82.2% had values lower than the 50th percentile, 55.6% lower than the 10th percentile, and 57.8% lower than the cut-off point for sarcopenia diagnosis.

Based on the classification for the SPPB, 42.2% were classified as disabled, 6.7% as frail, 24.4% as prefrail, and 26.7% as autonomous. Using the EWGSOP2 cut-off point, 69% exhibited impairment of functional status. However, no significant differences were found in strength or functional status according to the diagnosis of malnutrition.

### 3.3. Correlation between Nutritional Parameters

There was significant correlation between PA and anthropometric measurements (AMC: r = 0.53, *p* = 0.001; ASMI: r = 0.54, *p* < 0.001) and muscle strength (HGS: r = 0.54, *p* = 0.001).

BCM had a significant correlation with anthropometric measurements (AMC: r = 0.77, *p* < 0.001; CC: r = 0.58, *p* < 0.001; ASMI: r = 0.60, *p* < 0.001) and HGS (r = 0.69, *p* < 0.001).

**Figure 1:**
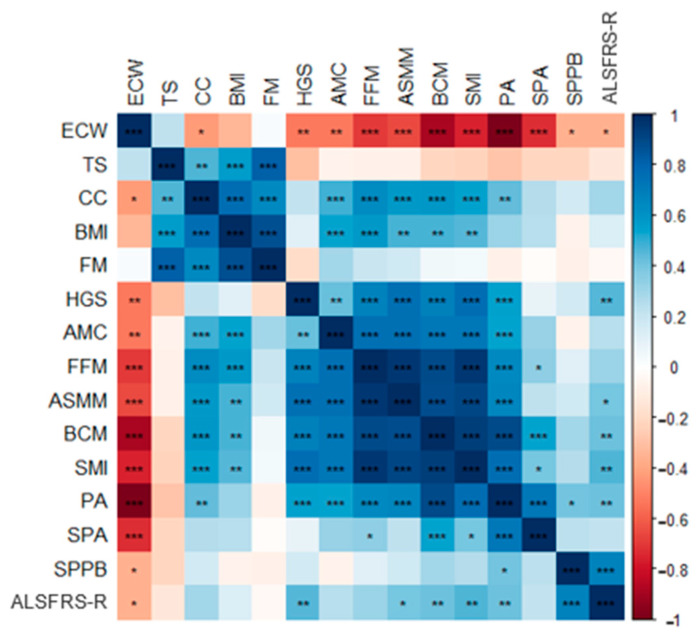
Pearson’s correlation plot of morphofunctional and disease evolution scores. Red colors indicate negative correlation, while blue colors positive correlations. Color intensity represents the strength of the correlation, with deeper shades signifying stronger relationships. Asterisks (*) indicate significant correlation between variables according to the Pearson’s correlation test (* *p* < 0.05; ** *p* < 0.01; *** *p* < 0.001).

Disease progression measured by ALSFRS-r showed a significant correlation with morphofunctional parameters such as the functional test SPPB (r = 0.67, *p*< 0.001), muscle mass values (ASMM: r = 0.40, *p* = 0.02; SMI: r = 0.46, *p* = 0.01; BCM: r = 0.49, *p* = 0.01), PA (r = 0.38, *p* = 0.01), and HGS (r = 0.46, *p* = 0.003). All these results are detailed in Figure 1.

### 3.4. Malnutrition Risk Factors and Related Morphofunctional Parameter Cut-Off Values

We found that BIVA variables were associated with malnutrition risk. Specifically, an increase in PA was associated with a 75% decreased risk, an increase in SPA was associated with a 77% lower risk, an increase in BCM was associated with a 40% decreased risk, and an increase in %ECW increased the risk of malnutrition by 30% (results are detailed in Table 3).

**Figure 2:**
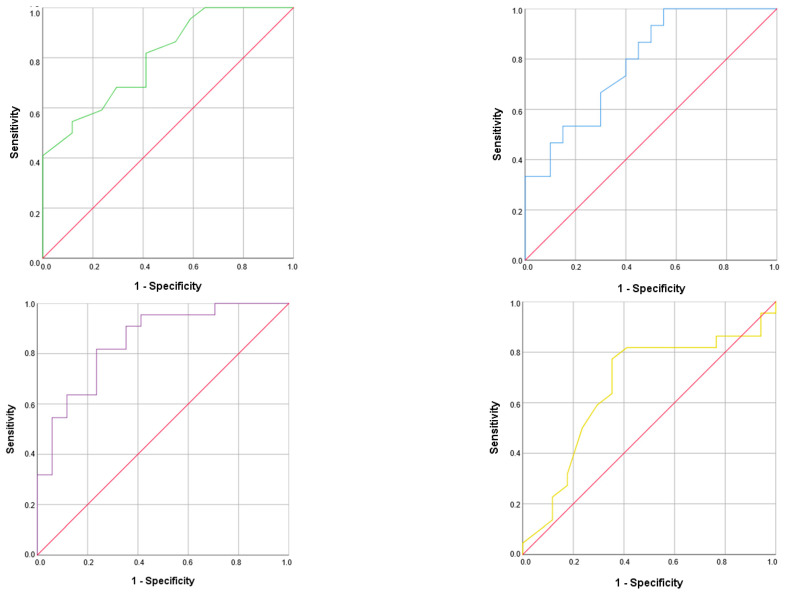
ROC curve of PA (**upside left**), SPA (**upside right**), ECW (**downside right**), and BCM (**downside left**).

The cut-off value obtained for the prediction of malnutrition for PA was 4.3° with an AUC of 0.801, a sensitivity of 82%, and specificity of 59%; the value for SPA was −0.8 with an AUC of 0.663, a sensitivity of 80%, and specificity of 60%; the value for ECW was 49.8% with an AUC of 0.787, a sensitivity of 93%, and specificity of 55%; and the value for BCM was 9.2 kg/m with an AUC of 0.850, a sensitivity of 96%, and specificity of 70% (Figure 2).

## 4. Discussion

This study showed a high prevalence of malnutrition (38%) in a sample of patients with ALS who were attended to at UMELA. According to GLIM criteria, malnourished ALS patients had lower PA and muscle and fat mass parameters. However, ECW values were higher in the malnutrition group. No significant differences were found in muscle strength and physical performance between both groups. PA was significantly associated with muscle strength and with muscle mass parameters measured by classic anthropometry. Additionally, PA was associated with worse disease evolution and physical performance. BIVA values such as PA, SPA, BCM, and ECW were independent prognostic factors for malnutrition in our sample.

The reported prevalence of malnutrition among ALS patients is heterogeneous, mainly due to diagnostic criteria used. According to the BMI, the malnutrition prevalence documented was 12% in a French ALS register of 117 patients (mean BMI: 24.6 ± 4.6 kg/m^2^) and 14.7% in a study performed in Brazil with 34 ALS patients (mean BMI: unknown) [22,23]. This differs from our results, as we found a higher malnutrition prevalence (38%) with a similar BMI (mean BMI: 25.2 kg/m^2^). Using the same criteria, a higher malnutrition prevalence (45%), has been described in a Brazilian study, which may be explained by a lower mean BMI (21.7 kg/m^2^) [24].

On the other hand, López-Gómez et al. studied the nutritional status of 93 ALS patients in Spain according to VGS and GLIM criteria, finding a higher prevalence when they considered VGS (71%) compared with GLIM criteria diagnosis (48%). Compared with these results, we detected a slightly lower prevalence (38% vs. 48%) with our sample having a comparable mean BMI (25.6 kg/m^2^ vs. 24.4 kg/m^2^) and mean age (65 vs. 67 years) [25]. Nakamura et al. found a 36% malnutrition prevalence in 48 Japanese subjects with ALS; defining it as % ideal body weight <0.9 [26]. Although there was a similar malnutrition prevalence, the results are not comparable because their sample had a lower mean BMI, the patients were older, and there was a higher proportion of females. Controversy remains concerning weight and BMI as useful tools to diagnose malnutrition in persons with ALS, due to the disease’s intrinsic characteristics. These include muscle atrophy and hypermetabolism, which complicate accurate assessments. Although the GLIM criteria are considered practical and comprehensive for diagnosing malnutrition across various clinical settings, their application in ALS requires further validation and reliable techniques. The criteria’s broad applicability and incorporation of multiple indicators provide a robust framework for assessing malnutrition, but they must be adapted to account for the unique challenges presented by ALS [27].

Beyond weight loss and BMI, we found significant differences between malnourished and non-malnourished patients in muscle mass parameters measured by anthropometry, with significantly lower values in malnourished patients in AC, AMC, and ASMI. These have been considered useful tools to detect malnutrition in ALS patients [28]. Salvioni et al. revealed a significant positive correlation between disease progression and AMC [24], supporting our results that muscle mass measurements are related to malnutrition and disease severity.

BIVA can indirectly estimate body composition, representing an useful, non-invasive, and validated assessment tool in people with ALS [9]. In our study, the muscle mass parameters measured by BIVA (FFM, BCM, ASMM, and SMI) showed significantly lower values in malnourished patients compared with those without malnutrition. Therefore, this tool could be useful for measuring active muscle mass and monitoring nutritional treatment in patients with. We also found lower FM in the malnourished group. Li et al. reported results which showed that patients with a significant weight loss (>5%) at diagnosis had a significantly lower BMI, FM, and FFM than those without weight loss [29].

Although in our study we detected a lower FM in the ALS malnourished group, no differences were found in the TS measurement. This could be due to the decreased visceral fat mass with no decrease in the subcutaneous fat mass. This is supported by a study by Choi et al. (2023) which analyzed using the subcutaneous fat volume index (FVI) and visceral FVI from abdominal CT, finding that visceral FVI declined during the disease progression while subcutaneous FVI did not [30].

When analyzing hydration status, in our sample, the malnourished group had a lower TBW and a higher ECW value and ECW/ICW ratio. Malnutrition often results in lower muscle mass, therefore the ICW is reduced as well, leading to an increase in the ECW [31]. The ratio ECW/BCM has been proposed as a potential biomarker for disease onset in pre-symptomatic ALS gene carriers [32]. Our results showed a negative correlation of ECW with muscle mass, functionality, and disease severity, suggesting that an increase in ECW might be a useful marker of malnutrition. It highlights the importance of evaluating changes in hydration status.

We used BIVA not only to assess body composition but also to evaluate body bioelectrical properties, reflected by PA [33]. PA has been associated with poor survival [22]. The PA mean value in our work was slightly lower than the one recently reported by López-Gómez et al. in a similar ALS sample [34]. Our sample showed lower PA values in the malnourished group compared to well-nourished people. Desport J.C. et al. presented similar results, although mean PA in both groups (malnourished and non-malnourished) was remarkably lower than ours [35]. This difference could be due to the higher disease severity of the patients, measured by ALSFRS-R, since our mean value was higher than theirs. Similarly, Barone M. et al. compared PA values between groups categorized by BMI, finding lower PA values in the underweight group compared to the normal weight group [36]. No differences in PA values were observed between spinal or bulbar onset groups [25].

In a recent study, PA and body composition were investigated as biomarkers, finding that pre-symptomatic ALS mutation carriers exhibited lower PA compared to non-carriers [32]. This finding underscores the potential of using body composition as a non-invasive tool for future biomarker research.

Despite BIVA not being universally regarded as the gold standard for morphofunctional assessment, it offers valuable insights into body composition and fluid distribution. However, it is important to recognize that other tools can provide complementary or more detailed assessments of morphofunctional status. One complementary tool widely used in morphofunctional assessment is Nutritional Ultrasound^®^. It has gained prominence for its ability to assess muscle and fat tissue directly [37]. In fact, a recent study found direct correlations between muscle mass parameters measured by ultrasonography (quadriceps thickness) and muscle mass markers from BIVA (e.g., BCMI, FFMI, ASMI) [34].

Our study showed a significantly positive correlation between PA and HGS. Consistent with this finding, the literature has evidenced the relationship between PA and muscle strength (HGS) in healthy adults, as well as in individuals with cancer, kidney disease, chronic obstructive pulmonary disease, and heart failure disease [38].

However, there is no existing evidence assessing muscle strength using HGS in subjects with ALS to compare with our results, since muscle strength in ALS patients has typically been evaluated using the Medical Research Council Scale [29,39] and knee extension strength [40]. We observed that the mean HGS value (20 ± 10.3 kg) was significantly lower compared to the cut-off points proposed by the EWGSOP2 [17]. Nevertheless, in studies from different populations, HGS has demonstrated an association with nutritional status [41]. We did not find these results in our sample. However, we found significant correlations with other nutritional values, such as muscle mass measurements.

We also found a correlation of HGS with ALSFRS-R. Supporting our results, ALSFRS-R has been correlated with muscle mass in other studies [29,30]. We assessed functionality with the SPPB, which has demonstrated its value as a predictor of all-cause mortality [42]. However, there is no evidence of the assessment in physical function using SPPB in ALS populations. In our sample, we found a high prevalence of impairment of physical function, with only 27% of patients with ALS showing a normal result in the test (SPPB > 10). In a study conducted by Montes J. et al. using a Timed Up and Go (TUG) test, an association was found between the TUG test and the risk of falls [43]. Furthermore, SPPB had a positive and significant correlation with ALSFRS-R and PA.

In the assessment of the capacity of morphofunctional tools as prognostic factors for malnutrition, we highlight the results of the ECW, whose increase raised the risk of malnutrition. In the case of BCM, FM, PA, and SPA, their decrease raised the risk of malnutrition. Additionally, we identified cut-off points for ECW (>49.75%), PA (<4.3°), SPA (<−0.8), and BCM (9.2 kg/m) that could distinguish malnourished ALS patients. These specific cut-off points within the GLIM criteria tailored for the diagnosis of malnutrition in patients with ALS. Traditional reliance on standard metrics like weight and BMI often fails to accurately reflect the nutritional status of ALS patients due to the disease’s characteristic muscle atrophy and altered metabolism.

To our knowledge, this is the first study that analyses body composition and its association with malnutrition diagnosed by GLIM criteria in ALS patients. Another strength of this study is that assesses not only nutritional status and body composition but also their interaction with functionality. Despite the absence of a standardized protocol for assessing the nutritional status of patients with ALS, guidelines recommend evaluating weight loss, and, if possible, body composition by DEXA or BIA, in addition to BMI. We used BIVA to assess body composition that has been validated in this population compared to gold standard body composition tools. However, this study has several limitations, such as the modest sample size and the differences in timing between nutritional assessment and diagnosis, which may lead to a heterogeneous sample. Moreover, it was conducted in a single center, and the results could be biased by our routine clinical practices. We also used general cut-off points for assessing nutritional status, as there is a lack of cut-off points specific for ALS. Therefore, it is important to register all clinical characteristics evaluated to compare our results within each clinical population.

## 5. Conclusions

Patients with ALS showed a significant impairment of nutrition status according to GLIM criteria. Malnourished ALS patients exhibited declines in body compartments, evidenced by lower muscle mass, lower fat mass, and higher extracellular water. Malnutrition was also associated with worse ALSFRS-R scores. Despite the deterioration of functional status, there were no differences in functional measurements according to malnutrition diagnosis. BIVA values such as PA, SPA, and ECW are useful for detecting malnutrition risk in ALS patients. Future lines of research are needed to test which cut-off points of morphofunctional tools best identify clinical outcomes and survival in ALS.

## Figures and Tables

**Table 1:** Clinical characteristics of the population according to malnutrition diagnosis.

	Total (*n* = 45)	Non-Malnutrition (*n* = 28/62%)	Malnutrition (*n* = 17/38%)	*p*-Value
Sex				0.02
Male	62.2	75.0	41.2	
Female	37.8	25.0	58.8	
Age (years)	65 ± 9.9	62 ± 10	70 ± 9	0.01
Disease evolution (months)	32 ± 27	37 ± 31	23 ± 19	0.15
Dysphagia diagnosis (%)	49	29	83	<0.001
PRG/PEG use (%)	15.6	3.6	35.3	0.01
NIMV (%)	20	14.3	29.4	0.40
ALSFRS-R (points)	32	34	30	0.02

Results are expressed as mean ± SD (numeric variables) or % (categorical variables). ALSFRS-R: Revised Amyotrophic Lateral Sclerosis Functional Rating Scale (0–48); NIMV: non-invasive mechanical ventilation; PEG: percutaneous endoscopic gastrostomy; PRG: percutaneous radiologic gastrostomy.

**Table 2:** Morphofunctional assessment variables and differences by malnutrition diagnosis.

	Total (*n* = 45)	Non-Malnutrition (*n* = 28, 62%)	Malnutrition (*n* = 17, 38%)	*p*-Value
**Anthropometric measures**				
Weight (kg)	69.3 ± 13.7	75.1 ± 10.2	59.6 ± 13.5	<0.001
Weight loss (%)	9.5 ± 8.2	5.8 ± 2.5	11.8 ± 9.6	0.03
BMI (kg/m^2^)	25.16 ± 4.64	27.4 ± 4.1	24.2 ± 2.8	<0.001
AC (cm)	27.8 ± 4	29.35 ± 3.7	25.4 ± 3.3	0.001
TS (mm)	15 ± 3.6	15.7 ± 7.7	13.8 ± 7	0.43
AMC (cm)	23.1 ± 3.4	24.3 ± 2.7	21 ± 3.4	0.001
CC (cm)	34.7 ± 3.6	33.3 ± 2.6	32.6 ± 2.3	0.39
ASMI (kg/m^2^)	6.4 ± 1.4	6.9 ± 1.4	5.6 ± 1.2	0.01
**BIVA**				
Rz (Ohm)	593.6 ± 106.1	549.8 ± 77.1	651.9 ± 113.6	0.01
Xc (Ohm)	48.6 ± 8.8	50.4 ± 9.2	46.2 ± 7.8	0.17
PA (°)	4.63 ± 0.96	5.09 ± 0.8	4.05 ± 0.8	<0.001
SPA	−0.85 ± 1.19	−0.55 ± 0.94	−1.26 ± 1.38	0.08
TBW (kg/m)	21.5 ± 3.4	23.1 ± 2.7	19.5 ± 3.3	0.001
ECW (%/TBW)	53.1 ± 6	50.3 ± 4.4	56.7 ± 6	0.001
ECW/ICW	1.17 ± 0.32	1.03 ± 0.18	1.36 ± 0.36	0.01
FFM (kg/m)	29 ± 4.4	31.2 ± 3.1	26.2 ± 3.8	<0.001
FM (kg/m)	11.9 ± 5.5	13.7 ± 5.7	9.5 ± 3.4	0.02
BCM (kg/m)	13.3 ± 3.6	15.2 ± 3.0	10.8 ± 2.8	<0.001
ASMM (kg)	17.4 ± 4.3	19.4 ± 3.4	14.8 ± 4.1	0.001
SMI (kg^2^/m^2^)	7.9 ± 1.7	8.7 ± 1.3	6.9 ± 1.6	<0.001
**Functional status**				
HGS max (kg)	20 ± 10.3	22 ± 9.8	17 ± 10.9	0.18
SPPB	5	7	4	0.19

Results are expressed as mean ± SD (numeric variables). AC: arm circumference; AMC: arm muscle circumference; ASMI: appendicular skeletal mass index; BMI: body mass index; BCM: body cell mass; BIVA: Bioimpedance Vectorial Analysis; CC: calc circumference; ECW: extracellular water; FFM: fat-free mass; FM: fat mass; HGS: hand grip strength; PA: phase angle; SMI: skeletal muscle index; SPA: standardized phase angle; SPPB: Short Physical Performance Battery; TBW: total body water; TS: tricipital skinfold.

**Table 3:** Logistic regression for the association between morphofunctional variables and malnutrition status.

	OR_c_ (95%CI)	*p*-Value	OR_adj_ (95% CI)	*p*-Value
Rz	1.01 (1.00–1.02)	0.01	1.01 (0.99–1.02)	0.07
Xc	0.94 (0.86–1.03)	0.17	0.94 (0.84–1.06)	0.36
PA	0.21 (0.07–0.60)	0.004	0.25 (0.08–0.80)	0.02
SPA	0.56 (0.30–1.10)	0.10	0.23 (0.06–0.90)	0.03
%ECW	1.30 (1.06–1.55)	0.01	1.23 (0.99–1.50)	0.051
BCM/h	0.60 (0.43–0.93)	0.02	0.60 (0.43–0.90)	0.001
SMI	0.39 (0.20–0.73)	0.004	0.39 (0.14–1.07)	0.07
FM/h	0.80 (0.70–0.94)	0.03	0.80 (0.60–0.98)	0.04
ALSFRS-R	0.92 (0.85–0.99)	0.02	0.93 (0.86–1.02)	0.12
SPPB	0.90 (0.80–1.04)	0.17	0.94 (0.80–1.10)	0.45
HGS	0.58 (0.04–4.50)	0.60	0.19 (0.01–2.01)	0.17

OR_c_: crude Odds Ratio, OR_adj_: Odds Ratio adjusted by sex and age. 95% CI: 95% confidence interval.

## Data Availability

The data presented in this study will be made available upon request to the corresponding author.
